# Challenges of Diagnosing Pseudohypoaldosteronism (PHA) in an Infant

**DOI:** 10.1155/2022/9921003

**Published:** 2022-07-11

**Authors:** Ghufran Saeed Babar, Minah Tariq

**Affiliations:** ^1^Children's Mercy Hospital and Clinics Endocrinology, 3101 Broadway Blvd, Kansas, Missouri 64111, USA; ^2^Kansas City University, College of Osteopathic Medicine DO Candidate, Class No 2024, Kansas, Missouri 64124, USA

## Abstract

*Background*. Pseudohypoaldosteronism (PHA) is characterized by renal tubular resistance to aldosterone. As a result, the symptoms typically involve hyperkalemia and hyponatremia. The aim of this clinical case report is to highlight the severe electrolyte imbalance PHA can present within an infant, as well as difficulties in diagnosing the condition. *Case Presentation*. A 5-week-old male arrived at the ER with episodes of emesis, lethargy, and difficulty in feeding. He had significant electrolyte abnormalities and was being treated by his PCP for failure to thrive. He presented with urinary sodium wasting, indicated by hyponatremia, hyperkalemia, low chloride, and hypercalcemia. Patient was treated with IVF and NaCl supplementation to normalize the electrolytes. The patient showed heterozygosity for a variant in the *WNK1* gene, which typically causes Gordon syndrome; however, our patient had a normal blood pressure. The electrolyte imbalance self-resolved during several months of follow-up, and currently, the patient is not on any treatment.

## 1. Introduction

Pseudohypoaldosteronism is a heterogeneous group of disorders with end organ resistance to aldosterone resulting in hyponatremia, hyperkalemia, and metabolic acidosis. The intravascular volume could be low or normal with a corresponding low or high blood pressure. The various forms of PHA can have elevated, normal, or low levels of renin and aldosterone, in the absence of elevated 17-OHP level [1, 2].

PHA can be further subcategorized into PHA types 1 and 2. PHA1 is a rare autosomal recessive disease caused by impaired transepithelial sodium transport that typically effects children and causes end organ resistance to aldosterone [3]. Patients with this disease do not benefit from mineralocorticoid treatment and must be given large doses of sodium supplementation to correct their symptoms. There are multiple mutations that can result in PHA1, which can either cause inactivation of the human mineralocorticoid receptor (MR, NR3C2 gene) in the kidneys or biallelic inactivation in the genes encoding the *α*, *β*, or *ɤ* subunits of the epithelial sodium channel (ENaC) [3]. Inactivation of the ENaC channels is typically caused by inactivation of *SCNN1A*, *SCNN1B*, or *SCNN1G* and results in systemic salt loss from the kidneys, salivary glands, colon, and sweat ducts [3]. Most children with the renal form of PHA1 are weaned off sodium supplementation relatively early due to maturation of the handling capacity of the kidneys [3]. However, patients effected by the systemic form of PHA1 often require life-long salt supplementation and have extensive medical needs [4].

There is a second form of PHA known as Gordon syndrome (PHA2) [5]. It characteristically presents as hypertension (HTN) and hyperkalemia with a normal glomerular filtration rate, acidosis, and a low plasma renin level. The aldosterone levels can be low to inappropriately high [6]. Gordon syndrome is predominantly an autosomal dominant condition with four genes that are implicated: *WNK1*, *WNK4*, *KLHL3*, and *CUL3* [7].

Young patients who present with the symptoms of hyponatremia and hyperkalemia are typically screened for congenital adrenal hyperplasia (CAH), as it is associated with PHA like electrolyte imbalance [8, 9].

## 2. Case Presentation

We report a case of a 5-week-old male presenting with severe salt-wasting, hyperkalemia, and resultant failure to thrive. The patient was born at 36 weeks with an uncomplicated vaginal delivery. The mother had preeclampsia and thus required induction of labor. He had an uncomplicated postnatal course and regained birth weight by 14 days, being fed primarily through breastfeeding. Initially, the patient was feeding well, but for 10 days, leading to his ED visit, he had developed a weight plateau, and he was transitioned to formula feeds. He had nonbloody, nonbilious emesis that was worsening. Patient was taking 3–3.5 ounces per feed every 3-4 h, but then it decreased to 1-2 ounces at a time. He was stooling 1-2/day and urinating 4-5/day. He was being followed by his PCP for failure to thrive. He had been placed on ranitidine to treat his reflux but had no significant improvement. Newborn screening had been done, but parents did not receive any notable results.

The patient was referred to the ED with increased difficulty in feeding, lethargy, and episodes of emesis. In the ED, the child was afebrile and in a compensated shock and had a low normal BP: 76/35, HR: 169/min, and fast breathing R/R; 80/min. Lab work completed for the patient showed Na of 112 mEq/L (132–142) and K of 10.1 mEq/L (3.5–6.2), calcium was 12.3 mg/dl (8.6–10.5), and CRP was normal. The urine test was normal and did not show signs of urinary tract infection (UTI). There is no previous history of UTI. Once endocrinology was consulted, patient was given a normal saline (NS) fluid bolus 20 ml/kg, and a repeat BMP was ordered that showed Na of 110 mEq/L and K of 9.3 mEq/L with normal LFTs (liver function tests). With continued salt imbalance, a second NS bolus was given and a dose of Florinef 0.2 mg was given due to suspicion of CAH. Subsequently, fluids were started with D5NS at 1.5 times maintenance for electrolyte and caloric replenishment. Follow-up electrolytes showed a stable Na of 109 mEq/L, a decreased K of 8.3 mEq/L, and a normal Ca of 10.2 mg/dl. Rest of the labs showed TSH level 5.3 mcIU/mL (0.35–7.60), free T4 2.2 ng/dL (0.8–1.9), random cortisol 20.5 mcg/dL (≥3), and a baseline 17-hydroxyprogesterone level of 99 ng/dl (normal).

The patient was admitted to the pediatric intensive care unit (PICU), was hemodynamically stable, and hydrocortisone was not started. Repeat labs were performed that showed an Na of 133 mEq/L, K of 4.7 mEq/L, Cl of 102 mEq/L, and a glucose level of 90 mg/dL. Considering a strong suspicion for CAH, a high dose ACTH stimulation test was performed. Results of this test showed a stimulated 17 OH-pregnenolone of 88 ng/dL, cortisol of 27.1 mcg/dL, and normal DOC (deoxy-corticosterone). His urine sodium level was elevated at 35 mmol/L (<20). The kidney and urinary system ultrasound were normal. Upon further evaluation, the patient's aldosterone levels were discovered to be elevated at 632 ng/dL (6.5–86) and renin level at 351 ng/dL (1.4–7.8). A diagnosis of PHA was made and the child stopped receiving Florinef, only receiving NaCl supplementation with an eventual dose of 22.5 mEq/kg/day to stabilize his electrolytes and compensate for his salt-wasting. He was discharged from the PICU in 6 days with a sodium level of 139 mEq/L.

Genetic testing was conducted on the patient and was negative for *NR3C2*, *CUL3*, *KLHL3*, *SCNN1A*, *SCNN1B*, *SCNN1G*, and *WNK4.* However, the patient showed heterozygosity for a variant of unknown significance, c.6276 T > A (*p*. Ser2092Arg) in the *WNK1* gene. Patient currently has normal electrolytes with salt supplementation and was gradually weaned off with PHA that has self resolved.

## 3. Discussion

Salt-wasting crisis presenting as a severe hyponatremia, hyperkalemia, and dehydration is life-threatening in an infant and requires urgent treatment. It could be caused by CAH (congenital adrenal hyperplasia), hypoadrenalism, congenital adrenal hypoplasia, isolated aldosterone deficiency, nephrotoxic medications (e.g., ACE inhibitors, nonsteroidal anti-inflammatory drugs) and pseudohypoaldosteronism [10, 11]. Like our patient, PHA can present as failure to thrive with symptoms of poor feeding, reduced weight gain, and vomiting [12]. However, infants can deteriorate and present with hypovolemic shock and life-threatening hyperkalemia, which can cause ventricular flutter [13, 14]. Our patient had elevated urine sodium level which indicated aldosterone deficiency or resistance; however, definitive diagnosis of PHA is made after the results of aldosterone levels and genetic tests.

Our patient showed elevated aldosterone level and a gene heterozygosity for *WNK1*, which is typically implicated in Gordon syndrome (PHA2) [2]. PHA2 is caused by molecular abnormalities in two *WNK* genes, including *WNK1* and *WNK4* [15]. Genetic defects in Kelch-like 3 (*KLHL3*) or Cullin 3 (*CUL3*) can also cause PHA2 [16]. However, our patient did not present with the typical hypertension which is present in Gordon syndrome due to an imbalance in electrolyte and acid–base equilibrium with increased circulating blood volume. Analysis of the patient's genetic study did not indicate any abnormalities in the genes that typically cause PHA1. Thus, the phenotype and genotype of this patient's transient illness did not correspond to the known presentations of PHA1 or 2, further making his case unique [17].

The patient's initial presentation to the ED of severe salt-wasting did not have any known precipitating events. There have been multiple accounts of pediatric PHA patients who show salt-wasting crisis after contracting a urinary tract infection (UTI) and is associated with obstructive uropathologies [18, 19]. These forms of PHA are frequently transient, like our patient's presentation, and present with a decreased glomerular filtration rate [20]. However, our patient did not have any known UTI prior to the onset of his weight loss and salt-wasting symptoms nor did he have a congenital obstructive uropathology.

## 4. Conclusions

There are several very interesting aspects about this case of transient PHA. First, it indicates that pseudohypoaldosteronism should be considered in any infant presenting with hyponatremia and hyperkalemia after the first few weeks of life, since congenital adrenal hyperplasia usually gets diagnosed by this time either by the results of the newborn screening or because the newborn has already presented with salt-wasting crisis. Second, our patient had a nonapparent cause of his symptoms and there were no obvious precipitating factors such as UTI or obstructive uropathies. Determining the exact cause of the onset of this patient's severe symptoms was difficult because the many subtypes of PHA have different presentations and genetic mutations. His genetic testing showed that he does not have any mutations in the genes that cause PHA1 specifically, yet he showed the corresponding symptoms of hyperkalemia, hyponatremia, and failure to thrive. Third, our patient showed the transient form of aldosterone resistance, distinguishing this condition from the known genetic causes of pseudohypoaldosteronism. The patient gradually recovered from the electrolyte imbalance and all medications, and later, salt supplementation was also discontinued. The child continues to have normal sodium, potassium, anion gap, and glucose levels. Furthermore, although the child does display heterozygosity for the *WNK1* gene which is typically implicated in Gordon syndrome (PHA2), he did not present with the characteristic hypertension of Gordon syndrome as well. Long term clinical follow-up will be required to determine if there any changes in electrolyte balance.
